# Rethinking pre-training: cognitive load implications for learners with varying prior knowledge

**DOI:** 10.3389/fpsyg.2025.1628047

**Published:** 2025-08-07

**Authors:** Anna Gorbunova, Anastasiia Kapuza, Ouhao Chen, Jamie Costley

**Affiliations:** ^1^HSE University, Moscow, Russia; ^2^School of Education, Faculty of Social Sciences, University of Leeds, Leeds, United Kingdom; ^3^College of Education, United Arab Emirates University, Al Ain, United Arab Emirates

**Keywords:** cognitive load theory, prior knowledge, pre-training, expertise reversal effect, redundancy effect, instructional design, concept mapping

## Abstract

This study examines how prior knowledge and pre-training relate to cognitive load during problem-solving. Grounded in cognitive load theory, it investigates whether pre-training facilitates learning by reducing cognitive load or imposes redundant information for learners with higher prior knowledge. In an experiment with 136 university students, pre-training was implemented through concept maps and a glossary introducing essential terms and procedures before problem-solving. Results revealed that learners with higher prior knowledge experienced lower intrinsic and extraneous load and higher germane load during problem-solving compared to learners with lower prior knowledge, suggesting enhanced schema refinement rather than redundancy. Pre-training consistently reduced extraneous load across all learners, including those with higher prior knowledge, challenging the expected expertise reversal effect. While learners with lower prior knowledge did not show significant reductions in intrinsic load, they benefited from decreased extraneous load during problem-solving. These findings highlight the value of pre-training as an instructional strategy and underscore the importance of aligning instructional design with learners’ existing knowledge.

## Introduction

Cognitive load theory provides a framework for understanding how human cognitive architecture influences learning by emphasizing the interaction between working memory and long-term memory (Sweller et al., 2019). According to the theory, learning is optimized when cognitive load is effectively managed, ensuring that intrinsic cognitive load (ICL) is within manageable limits, extraneous cognitive load (ECL) is minimized, and germane cognitive load (GCL) is maximized to facilitate schema construction (Paas et al., 1994; Sweller, 2010).

Prior knowledge is widely recognized as a crucial factor in reducing ICL (Sweller, 2024). Experienced learners rely on pre-existing schemas, which allow them to integrate new information more efficiently, reducing the number of interacting elements (i.e., “element interactivity” discussed in details below) that must be processed simultaneously (Kalyuga, 2007a, 2007b; Sweller et al., 1998) through chunking multiple elements into larger meaningful units, thereby increasing working memory capacity (Paas and Van Merriënboer, 2020). However prior knowledge does not always reduce ICL; in some cases, learners with greater expertise having more refined schemas, may perceive tasks as more complex, while novices often underestimate difficulty and report lower mental effort (Endres et al., 2023; Zambrano et al., 2019). On the other hand, learners with more prior knowledge have more developed schemas and may thus have more working memory available (Paas and Van Merriënboer, 2020). This allows them to better manage suboptimal instructional design and indirectly affect ECL.

Prior knowledge activation – the process of prompting learners to retrieve and apply relevant knowledge before engaging with new instructional content – is a key strategy for mitigating ECL and optimizing learning efficiency (Glogger-Frey et al., 2022; Schwartz and Martin, 2004). Effective prior knowledge activation can help learners connect unfamiliar concepts with existing schemas, improving comprehension and facilitating deeper learning (Retnowati et al., 2018). Various instructional techniques, such as concept mapping (Gurlitt and Renkl, 2010), worked examples (Zambrano et al., 2019), and supplying relevant prior knowledge to ease the processing of information (Mayer and Pilegard, 2014), have been proposed as methods for activating prior knowledge. However, the effectiveness of these techniques depends on the level of learners’ prior knowledge (Sinha and Kapur, 2021).

Explicit prior knowledge activation methods aim to cue relevant existing knowledge, without introducing new content. While novice learners benefit from them, learners with higher prior knowledge may experience the expertise reversal effect, when instructional guidance becomes redundant and increases ECL (Kalyuga et al., 2003; Sweller et al., 2011).

A key distinction must be made between prior knowledge activation and pre-training, as they serve different instructional purposes. Prior knowledge activation encourages retrieval of what the learner already knows without introducing entirely new content (Gurlitt and Renkl, 2010; Glogger-Frey et al., 2022). Pre-training, in contrast, provides new but foundational information before the main learning activity, often to reduce ICL and prepare learners for more complex tasks (Mayer and Pilegard, 2014). Although pre-training is not designed to activate prior knowledge, it may incidentally do so, especially for learners who already possess some familiarity with the topic. This dual role is particularly relevant when considering the differences in learners’ prior knowledge. In this context, distinguishing between pre-novices (learners with no background knowledge in a specific domain) and novices (learners who possess only minimal domain-specific knowledge) becomes important (Gorbunova et al., 2023). It also relates to the expertise reversal effect, which occurs when instructional support that helps novices becomes redundant or even disruptive for experts (Sweller et al., 2011; Kalyuga, 2007a, 2007b). Therefore it is essential to consider whether pre-training in this case might also help to activate prior knowledge or lead to redundancy for learners with higher knowledge due to the expertise reversal effect.

This study investigates the relationship between prior knowledge, pre-training, and cognitive load. By examining this relationship, this study seeks to better understand the role of pre-training in cognitive load management and contribute to the ongoing discussion regarding adaptive instructional design for learners with varying levels of expertise.

## Literature review

### Cognitive load theory

Cognitive load theory provides a framework for understanding how human cognitive architecture influences learning by emphasizing the interaction between working memory and long-term memory (Sweller et al., 2019). Working memory has severely limited capacity and can only process a small number of new elements at any given time (Miller, 1956; Sweller, 2010). In contrast, long-term memory serves as an extensive storehouse of knowledge, where information is structured as schemas that allow individuals to categorize and integrate information efficiently (Paas et al., 1994). When learners encounter new materials, they must process them within the constraints of working memory before it can be encoded into long-term memory. Schema construction and automation allow large amounts of information to be stored in long-term memory and processed as cohesive units in working memory, which not only enhances cognitive efficiency but also reduces working-memory load by minimizing the need for conscious processing of individual elements (Kirschner, 2002).

Cognitive load theory distinguishes three types of cognitive load: intrinsic, extraneous, and germane (Sweller et al., 2019). IICL is inherent to the material itself and depends on the level of element interactivity (i.e., the elements must be simultaneously processed and understood in relation to each other in the working memory) (Sweller, 2010; Ayres, 2006). ECL, on the other hand, is imposed by poor instructional design and does not contribute to learning (Sweller et al., 2011). Inefficient presentation formats, redundant information, and split-attention information where learners must integrate information from multiple sources can overload working memory and hinder schema construction (Chen et al., 2018). Importantly, ICL and ECL types are additive and thus minimizing ECL frees up working memory capacity, allowing more resources to be allocated to processing ICL (Paas and Van Merriënboer, 2020).

In contrast, GCL refers to the mental effort invested in creating and refining schemas, which facilitates long-term retention and transfer of knowledge (Paas et al., 2003). Unlike ICL and ECL, GCL is not an independent type of load but rather a function of the cognitive resources allocated to relevant learning processes (Sweller et al., 2019). Effective instructional design aims to minimize ECL while optimizing GCL to promote deep learning (Sweller et al., 2019). Effective learning occurs when cognitive load is managed to avoid exceeding the limits of working memory while maximizing schema construction and automation (Sweller et al., 2019).

The concept of element interactivity is central to cognitive load theory (Chen et al., 2023). Element interactivity refers to the degree to which elements of information must be processed simultaneously, and it fundamentally determines ICL. High element interactivity imposes a greater burden on working memory, making learning more challenging (Sweller, 2010). Element interactivity also estimates learning complexity based on the number of elements and their degree of interaction imposed on working memory (Chen et al., 2024). Tasks with high element interactivity, such as solving complex problems with multiple interdependent components that must be processed simultaneously, impose a heavier cognitive burden than tasks with low element interactivity, where elements can be learned independently and separately (Chen et al., 2017). For instance, memorizing basic definitions of programming terms, such as “variable,” “loop,” or “function,” represents a low element interactivity task because each concept can be learned independently without requiring connections between them. In contrast, writing a working computer program requires processing multiple interrelated concepts simultaneously, such as control structures, data types, and syntax rules, making it a high element interactivity task.

However, element interactivity also is associated with ECL when instructional design introduces unnecessary cognitive burden due to suboptimal instructional design (Sweller, 2010; Chen et al., 2023). For example, as discussed by Chen et al. (2023), when learning materials are high in element interactivity, worked examples are more effective than unguided problem solving. Worked examples reduce cognitive search, allowing learners to focus on understanding interactions between elements. In contrast, requiring novices to solve complex problems without guidance forces them to process many interactive elements while generating solutions, which can overload working memory. The prior knowledge of learners serves as a moderator to the levels of element interactivity so does to the levels of cognitive load.

Problem-solving has been conceptualized as a continuum ranging from routine to complex, with tasks differing in structure, familiarity, and cognitive demands. Thus problem-solving is not limited to ill-structured scenarios; tasks that involve applying conceptual knowledge, making distinctions, and constructing explanations also qualify as meaningful problem-solving activities (Jonassen, 2000). Moreover, for novice learners, well-structured problems that rely on established concepts (so-called “strong” methods) can effectively foster schema development while managing cognitive load (Van Merrienboer, 2013). For example, rule-based tasks such as calculating time zone differences, solving weekday offset problems, or transferring procedural knowledge in programming contexts all require structured reasoning and integration of multiple information sources demonstrating how well-structured tasks can effectively engage learners in meaningful problem-solving (Jo and Kim, 2020; Margulieux and Catrambone, 2021; Schmeck et al., 2015). This study builds on these frameworks by examining how structured, conceptually demanding tasks engage learners in foundational problem-solving processes.

#### Prior knowledge and cognitive load

Prior knowledge refers to the existing subject-specific understanding that learners possess before engaging in a particular instructional experience (Gurlitt and Renkl, 2010). Prior knowledge stands as a fundamental prerequisite for effective learning and plays a critical role in mitigating ICL (Sweller, 2024). It is assumed that experienced learners having more advanced prior knowledge can rely on pre-existing schemas to reduce the number of interacting elements they must process in the working memory (Kalyuga, 2007a, 2007b), thus reducing the level of ICL. Because schemas reduce the number of interacting elements that must be processed individually by chunking, prior knowledge enables learners to allocate more working memory resources to new learning tasks (Sweller et al., 1998; Sweller, 2024) by reducing the cognitive load. Thus the availability of domain-specific schemas in long-term memory allows learners to effectively process new information and construct enriched schemas (Sweller et al., 2019). Prior knowledge also guides learners’ attention toward relevant information while minimizing distractions. Moreover, existing mental frameworks serve as organizational structures that facilitate the integration of novel information (Gurlitt and Renkl, 2010).

Although it is commonly assumed that the development of more advanced schemas reduces ICL, recent research suggests a more nuanced relationship between prior knowledge and cognitive load. For example, in complex problem-solving scenarios, expert learners may consider a broader range of additional factors that novices do not know or overlook (Endres et al., 2023), so they will end up processing more interacting elements. This will result in experts perceiving tasks as more complex leading to an increase in ICL. Another issue highlighted by previous research, in terms of prior knowledge, is self-assessment. It is hypothesized that the lack of prior knowledge may influence learners’ perception of task complexity, leading them to overestimate their performance and, in turn, report lower levels of mental effort (Zambrano et al., 2019). Thus, less knowledgeable learners may not fully grasp the complexity of a task, causing them to assess it as easier than it actually is. In contrast, learners with greater prior knowledge are more likely to recognize the number of interacting elements involved, leading to a more accurate perception of task difficulty and, in some cases, an increased sense of cognitive load.

From the cognitive load theory perspective, it is well-established that prior knowledge is crucial for managing *ICL*: the more prior knowledge a learner has, the lower the ICL is Sweller et al. (2019). Learners with more prior knowledge can rely on existing schemas to integrate and process interactive elements more efficiently, thereby reducing ICL (Kalyuga et al., 2003). Conversely, learners with less prior knowledge lacking such schemas experience greater difficulty managing too many elements, which can lead to cognitive overload. There could also be an indirect effect of prior knowledge on *ECL* since the more prior knowledge learners have, the more working memory resources they can allocate to deal with suboptimal design of learning materials that cause ECL. Prior knowledge can assist learners in effective processing of poorly designed instructional materials without overwhelming their working memory capacity (Sweller et al., 2011). As for *GCL*, that has a redistributive function from extraneous to intrinsic aspects of the task, it should increase along with the increase of prior knowledge (Sweller et al., 2019), as GCL has been characterized as an indicator or reflection of the underlying understanding that learners have developed within a given domain (Leppink et al., 2014).

While prior knowledge can reduce ICL by enabling more efficient schema-based processing, it may also influence ECL and GCL in complex ways. One way to leverage prior knowledge effectively in learning environments is through prior knowledge activation, a strategy designed to help learners retrieve and apply existing knowledge before engaging with new materials.

#### Prior knowledge activation

Activating prior knowledge can play a crucial role in preparing learners for subsequent learning. By connecting new information to learners’ existing knowledge and experiences, educators can create a link between unfamiliar content and more recognizable ideas, making the learning process more approachable and meaningful (Glogger-Frey et al., 2022; Gorbunova et al., 2023; Schwartz and Martin, 2004). The primary objective is to stimulate learners’ prior notions and intuitions, which may encompass partially relevant or even inaccurate prior knowledge (Kalyuga and Singh, 2016). However research highlights that while prior knowledge activation can yield several benefits, such as focusing on essential features during subsequent instruction, these advantages are conditional on the relevance of the activated knowledge, therefore, the situations when prior knowledge activation is high but its relevance to the learning task is low should be avoided (Sinha and Kapur, 2021). Previous research shows that providing prior knowledge for complex tasks led to significant improvements in performance outcomes (Retnowati et al., 2018).

Certain knowledge can be activated spontaneously, by reading and comprehending the introduction of the topic, while other knowledge is better established through instructional interventions that intentionally activate schemas (Gurlitt and Renkl, 2010). Instead of presenting new concepts or procedures in advance, prior knowledge activation focuses on drawing out and engaging learners’ existing mental representations to support the construction of new understanding. This distinction is particularly important for novice learners who may lack domain-specific schemas in long-term memory, and may therefore struggle to establish meaningful connections with provided information, so the instruction may be beyond their current level of comprehension, leading to confusion and frustration. In such cases, tasks that intentionally activate relevant prior knowledge become crucial (Gorbunova et al., 2023).

Instructional strategies aimed at prior knowledge activation often involve analogies or metaphors from other domains, brainstorming activities, enabling learners to construct new understanding that links learners’ experiences to upcoming concepts (Gorbunova et al., 2023).

Prior knowledge can be also activated through prompting learners to retrieve relevant information from long-term memory, which can enhance comprehension by linking new content to existing schemas. While prior knowledge activation focuses on recall, pre-training serves a complementary but distinct role in instructional design. It is assumed that learners benefit from being introduced to essential terminology prior to engaging with the core learning materials (Mayer, 2017). By establishing a solid foundation of understanding through the introduction of key concepts and contextual information, educators can facilitate learners’ comprehension and integration of new materials (Krieglstein et al., 2023).

Several empirical studies have illustrated the varied implementations of prior knowledge activation. For example, Zambrano et al. (2019) provided learners with a booklet containing conceptual explanations, a worked example, and practice problems on break-even point calculations. This phase aimed to reactivate and reinforce partially developed schemas rather than introduce entirely new content, enabling learners to draw on existing mathematical knowledge as a foundation for subsequent learning. This step was designed not only to activate relevant prior knowledge but also to structure the subsequent learning tasks effectively. By ensuring that learners first engaged with familiar operations in a new context worked examples before transitioning to problem-solving, the intervention helped students prepare for more complex tasks and reduced the likelihood of cognitive overload.

The productive failure (Sinha and Kapur, 2021) illustrates another approach to activate prior knowledge. During the productive failure learners are encouraged to generate their own solutions at the early stages of learning, even if they are incorrect or suboptimal. Thus this approach can serve as an essential catalyst for deeper knowledge integration. The activation of relevant but incomplete knowledge allows instructors to guide students in recognizing conceptual limitations, fostering a more refined understanding.

Finally, Gurlitt and Renkl (2010) explored the effectiveness of concept maps as a tool for prior knowledge activation, drawing on Ausubel’s (1968) theory of meaningful learning. In their study, two types of concept maps were tested, revealing that partially completed maps led to higher self-efficacy and improved learning outcomes compared to fully completed versions. These findings suggest that intentional gaps in information provided during prior knowledge activation can encourage deeper cognitive engagement and better integration of new information.

#### Pre-training

As students have different levels of prior knowledge, the instruction should be tailored to meet the needs of individuals, however it is not always realistic in authentic learning environments. For this reason, strategies should be adopted to mitigate the differences in prior knowledge before learners engage in key instructional activities. Such strategies are particularly important when using cognitively demanding problem-solving first methods for learners with different levels of prior knowledge. One potential way of levelling learners’ prior knowledge could be pre-training.

Pre-training refers to a learning intervention designed to develop learners’ specific knowledge and skills relevant to the target material, before they encounter the core learning content (Sweller et al., 2011), or the introduction of individual components before presenting the complete process (Clark et al., 2006). It serves to moderately enhance learners’ expertise without causing a significant leap in their skill or knowledge levels. According to cognitive theory of multimedia learning (Mayer, 2017), there is the pre-training principle when learners achieve better learning outcomes if they are familiar with the key features of the concepts (Mayer and Pilegard, 2014). Pre-training enables learners to construct an initial mental model that facilitates the integration of new information during subsequent learning (Mayer, 2002). However, the complete understanding of pre-training lacks clarity, and it’s important to maintain a clear distinction between pre-training and the actual learning process, ensuring that pre-training only provides essential knowledge without overlapping with the core instructional content (Meyer et al., 2019).

Pre-training is mostly aimed at reducing element interactivity so decreasing *ICL*, especially for novice learners (Sweller et al., 2011). When learners encounter a complex topic with high element interactivity, they must hold and manipulate multiple interacting elements at once, which may overload the working memory capacity. Pre-training helps reduce cognitive load by first teaching individual elements separately before combining them. For example, in the study of Retnowati et al. (2018) pre-training was designed as worked example booklets on two prerequisite topics. The materials aimed to establish topic-specific schemas in long-term memory providing a cognitive foundation for solving later tasks that required integrating both concepts. This approach, known as the isolated-interactive elements effect, improves learning by allowing students to understand parts in isolation before managing them together (Blayney et al., 2010). But pre-training could also indirectly interact with *ECL* by enhancing learners’ ability to self-manage their cognitive load.

When ICL is reduced through pre-training, learners have more available working memory resources, which can help them allocate cognitive effort more efficiently (Sweller et al., 2011). When learners perceive a task as less overwhelming, they are better able to control their cognitive resources, filter irrelevant information, and focus on essential aspects of learning. While pre-training does not directly eliminate ECL, the increase in available cognitive resources for schema acquisition may result in a lower perceived ECL (Krieglstein et al., 2023). ECL generally has a negative correlation with GCL (Costley et al., 2023), therefore with the decrease of ECL, more resources can be devoted to dealing with ICL and thus *GCL* will increase. Another key outcome of pre-training is the successful incorporation of new content elements into learners’ existing schemas, which positively affects GCL (Sweller et al., 2011). With pre-training, learners start the primary learning with some fundamental concepts acquired, ensuring that working memory resources are directed toward schema formation rather than managing instructional complexity (Sweller et al., 2019).

Pre-training was shown as an effective method to reduce unnecessary cognitive load (Jung et al., 2021). Although some studies show a strong positive effect of pre-training on skill transfer (Liu et al., 2021), there is controversial evidence suggesting that while ICL can be reduced through pre-training, it does not always lead to significant knowledge gains (Akbulut et al., 2024). Some studies have also shown that null effects of pre-training may be attributed to specific conditions that limit the utility of pre-training, such as high level of learners’ prior knowledge, since learners already know the information covered during pre-training (Mayer and Pilegard, 2014), as well as situations where it is more beneficial to present pre-training material concurrently with the main content. There are also cases when the material itself does not require substantial mental effort, for example the inherently simple or low element interactivity content that learners can directly engage with without exceeding their working memory capacity, thus pre-training becomes unnecessary (Meyer et al., 2019).

It implies that for learners with different levels of prior knowledge, pre-training should have different effects in terms of cognitive load. In a recent study of Jung et al. (2024), the purpose of introducing pre-training prior to problem-solving was to help novice learners to overcome insufficient self-regulation skills for grasping important principles or low self-efficacy that may hinder learning progress. However, while novice learners often benefit from supplementary information and guidance provided during instruction, it can be less effective or even detrimental when applied to more experienced learners, which is known as the expertise reversal effect (Chen et al., 2017). Thus on one hand, pre-training might serve as prior knowledge activation for learners with low prior knowledge, on another hand pre-training might induce the redundancy effect for learners with high level of prior knowledge (Sweller et al., 2011). It is therefore crucial to further explore and understand the effect of pre-training on learners with varying levels of prior knowledge.

While both prior knowledge activation and pre-training aim to prepare learners for subsequent learning, they differ in purpose and mechanism. Prior knowledge activation involves prompting learners to retrieve existing mental models, helping them connect new content to what they already know. In contrast, pre-training is used when learners lack relevant schemas and need foundational knowledge introduced explicitly. Although pre-training may incidentally activate prior knowledge, its primary goal is schema construction rather than retrieval.

#### Redundancy effect

The redundancy effect occurs “when material that includes redundant information results in less learning than the same material without the redundant information” (Sweller et al., 2011, p. 142). However, researchers distinguish between the same and unnecessary information, suggesting that the key difference is relevance. The same information supports learning by repeating relevant content, while unnecessary information, like background music, is unrelated to the task (Trypke et al., 2023). Nonetheless, the redundant information consumes limited working memory resources, diverting attention away from the primary learning task and hindering the retention of relevant information (Mayer and Moreno, 2003).

Recent research distinguishes between two types of redundancy: modal (or working memory channel) redundancy and content redundancy (see Trypke et al., 2023). Modal redundancy refers to the presentation of identical content across different sensory modalities, such as simultaneous text and narration or image with explanatory audio (Trypke et al., 2023). Content redundancy occurs when the same information is presented multiple times within the same modality (e.g., written text with an identical spoken narration or redundant visual labels). Findings on the effects of content redundancy are contradictory. This redundancy often increases ECL as learners must process repetitive information that does not contribute to deeper understanding (Trypke et al., 2023). At the same time, Albers et al. (2023) found that content redundancy might enhance learning outcomes and reduce cognitive load, but they acknowledge that their study did not account for prior knowledge, which may critically influence how redundancy is processed across different learner groups.

Prior knowledge plays a significant role in moderating the content redundancy effect (Trypke et al., 2023). For novices, content redundancy may still be essential for learning. Novices benefit from receiving the same information in multiple formats because this redundancy supports their understanding and reduces ICL. It compensates for missing knowledge structures, and helps them gradually integrate new knowledge into their developing schemas (Kalyuga, 2009). However, as learners acquire expertise, this redundancy becomes unnecessary and introduces ECL (Sweller et al., 2011). Experts, with their well-developed mental models and schemas, can process complex information without the need of multiple repetitions.

Therefore, instructional design must be tailored to the learner’s level of expertise: what is redundant for experts may still be necessary for novices to build an understanding (Sweller, 2010). The presence of content redundancy can exacerbate cognitive overload in expert learners by causing them to divert their limited cognitive resources toward processing irrelevant information, ultimately reducing learning efficiency and leading to expertise reversal effect (Sweller et al., 2011).

#### Expertise reversal effect

One of the consequences of the redundancy effect is the expertise reversal effect (Sweller et al., 2011, p.155). The expertise reversal effect occurs when instructional techniques that are beneficial for novices, such as highly detailed explanations and explicit guidance, become redundant or even counterproductive for more knowledgeable learners who may find them distracting (Kalyuga et al., 2003). This effect is driven by prior knowledge: learners with high prior knowledge are able to process new information more efficiently because they have developed well-structured mental schemas (Choi et al., 2014). Therefore, for expert learners, detailed instruction that benefits novices can become redundant, often leading to cognitive overload instead of facilitating learning (Kalyuga, 2009). In contrast, novice learners, lacking prior knowledge, require detailed instructions to build mental models and reduce ECL (Kalyuga et al., 2003).

Pre-training allows novice learners to acquire concepts before engaging in complex learning tasks, effectively reducing ICL during problem-solving (Mayer, 2002). By introducing key principles and relevant schemas early on, pre-training prepares learners to process more advanced material with greater efficiency. However, for expert learners, pre-training may contribute to content redundancy, as they may already have possessed the necessary background knowledge. Previous research shows that pre-training was particularly beneficial for learners with low levels of prior knowledge (Mayer and Pilegard, 2014). In such cases, an adaptive instructional approach, providing timely and relevant instructional guidance, where the depth and complexity of pre-training materials are adjusted based on prior knowledge, can help to minimize ECL and prevent the negative consequences of the expertise reversal effect (Kalyuga, 2007a, 2007b).

### This study

The interaction between prior knowledge and different types of cognitive load remains underexplored in the context of pre-training. Pre-training can facilitate learning by providing foundational knowledge that supports schema construction, but it may also hinder learning for more knowledgeable learners if it introduces redundant information. However, its effectiveness may vary, based on learners’ prior knowledge levels. Previous studies have focused on designing effective pre-training for novice learners. For example, Jung et al. (2024) examined how different types of pre-training enhance self-regulation and problem-solving for novices, focusing on scaffolding learning before cognitively demanding tasks. This study, however, investigates how pre-training influences intrinsic, extraneous, and GCL across different expertise levels. More specifically, it examines whether pre-training supports learning across all levels of prior knowledge or becomes redundant for learners with higher prior knowledge, offering a broader perspective on its role in cognitive load management.

*RQ1:* What differences in the cognitive load experienced by learners with varying levels of prior knowledge during problem-solving after pre-training?

*H1a:* Learners with higher prior knowledge will experience lower ICL compared to learners with lower prior knowledge.

*H1b:* Learners with higher prior knowledge will experience lower ECL compared to learners with lower prior knowledge.

*H1c:* Learners with higher prior knowledge will experience higher GCL compared to learners with lower prior knowledge.

While pre-training is often intended to support learning, its role in cognitive load management remains complex. For learners with lower prior knowledge, pre-training may help by providing essential foundational information thereby reducing ICL during problem-solving. In contrast, for learners with higher prior knowledge, pre-training may introduce redundant information, thereby increasing ECL, potentially disrupting schema-based processing and undermining learning rather than facilitating it (Kalyuga, 2007a, 2007b). This disruption could, in turn, reduce the resources available for germane cognitive processing. This study examines how pre-training interacts with prior knowledge levels to optimize cognitive load during the problem-solving stage, compared to pre-training stage.

*RQ2:* How does pre-training contribute to the redundancy effect for learners with different levels of prior knowledge during problem-solving?

Based on literature review, we expected different cognitive load outcomes for learners with varying levels of prior knowledge when pre-training is introduced:

*H2a:* For learners with lower prior knowledge, cognitive load during problem-solving will differ from the pre-training phase: ICL and ECL will be lower, while GCL will be higher during problem-solving compared to the pre-training phase.

*H2b:* For learners with higher prior knowledge, pre-training will be redundant, resulting in higher ECL, lower ICL, and lower GCL during problem-solving compared to the pre-training phase.

## Methodology

### Participants and context

To calculate the required sample size, we conducted an *a priori* power analysis using the *pwrss* package in R (Bulus, 2023), which is specifically designed for factorial ANOVA designs. A recent study by Jung et al. (2024), which examined the effects of different types of pre-training on cognitive load in programming education, reported effect sizes ranging from *η^2^* = 0.06 to 0.09. Based on this, we assumed an expected η^2^ of 0.06 (corresponding approximately to Cohen’s *f* = 0.25), which reflects a small-to-medium effect. Given a 2×2 mixed ANOVA design, a significance level of *α* = 0.05, and a desired power of 0.80, the analysis indicated that a total sample size of *n* = 125 would be required to detect main and interaction effects.

The participants in this study were second-year graduate students from various majors at a selective Russian university. A total of 136 students (73.6% women) from over 15 academic programs across various departments participated in the study during a synchronous online lecture as part of two elective education-focused courses. The students were told that they would be working with experimental materials designed to enhance their learning experiences, but they were not informed about the specific aims or details of the study. Instead, they were briefed that the instructional materials would follow an unusual format and that their insights on them as students in education would be particularly valuable for the study. The selected topic for instructional materials was “Cognitive Load”.

### Materials

Concept maps are one of the visual methods for activating prior knowledge (for review see Hattan et al., 2024). Such visual representations help learners see how their prior knowledge on concepts and subconcepts related to each other. Moreover, research suggests that pre-constructed concept maps are more effective as pre-training for learners with lower prior knowledge than having them create their own (Gurlitt and Renkl, 2008; Gurlitt and Renkl, 2010).

For this study, we developed pre-constructed concept maps focusing on the topic of “Cognitive Load” (see Supplementary materials). The maps included core concepts (e.g., intrinsic, extraneous, and germane cognitive load) and their interconnections. Additionally, brief explanatory labels and definitions were included to help guide learners through the structure without overwhelming them.

In addition to the concept maps, learners were provided with a glossary containing definitions of key terms related to cognitive load (Supplementary materials). The pre-training phase included both a concept map and a glossary. By providing pre-training, we aimed to support schema construction for learners with lower prior knowledge and test content redundancy for learners with higher prior knowledge. The glossary was intended to provide terminological clarity by defining key concepts, while the concept map was used to visually represent the relationships between these concepts. Although there was some content overlap between the two components, they served complementary functions and were designed to be used together as a unified tool.

### Design and procedure

**Figure 1:**
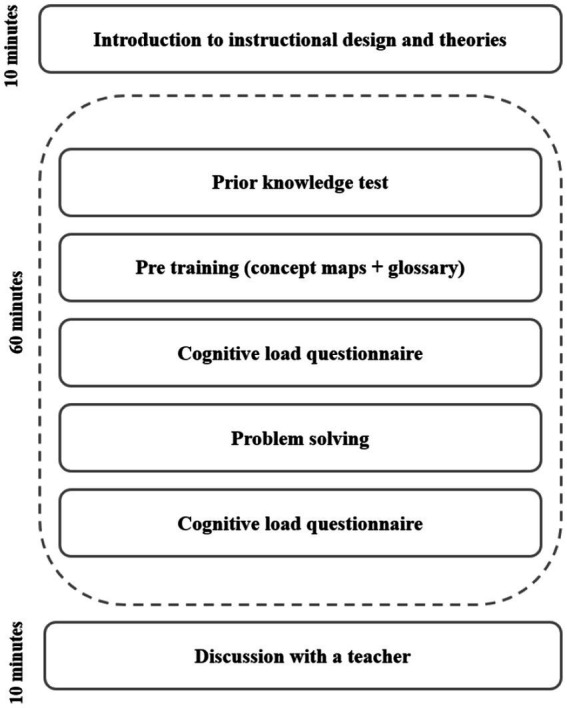
An overview of the procedure.

The procedure took 80 min to complete (Figure 1). First, the instructor briefly introduced the topic of instructional design and learning theories, as well as explained what concept maps are (approximately 10 min). After the briefing, each student received a unique link to Microsoft Forms. The forms were organized as follow (see Supplementary materials for questionnaire):

1. Prior knowledge test with multiple choice questions (appr. 7 min)
2. Pre-training: short introduction to work with concept maps and a concept map about cognitive load theory, a glossary with main definitions (appr. 7 min)
3. Cognitive load questionnaire measuring different types of cognitive load for using a concept map and glossary—pre-training (appr. 7 min)
4. Problem-solving activity (learners were able to use concept map and the glossary as instructional support) (appr. 30 min)
5. Cognitive load questionnaire measuring different types of cognitive load for problem-solving activity (appr. 7 min)

### Measurement

#### Prior knowledge

The prior knowledge test consisted of 8 multiple-choice questions covering various aspects of cognitive load (see Supplementary materials). Each correct answer was awarded 1 point, while incorrect answers received 0 point. The internal consistency of the test was evaluated using the Cronbach’s alpha with result (ɑ = 0.44) that is expected for short, dichotomously scored knowledge tests (Taber, 2018), and therefore do not necessarily meet the assumptions of item parallelism and unidimensionality (Edelsbrunner et al., 2025).

Next, we divided the students into two groups: lower prior knowledge and higher prior knowledge. Since the test was relatively easy with a mean of 6.3, we used the median score of 6 out of 8 as the cutoff^1^. Students who scored less than 6 points were classified as the lower prior knowledge group (26.5%), while those who scored 6 or higher were assigned to the higher prior knowledge group (73.5%). While the group sizes were unequal, regression models can reliably estimate effects under such conditions: it may reduce statistical power for the smaller group, but do not bias the overall results (Zhan et al., 2021). Moreover, the groups showed significant differences in ICL during both the pre-training and problem-solving stages, confirming that they represent distinct levels of prior knowledge.

#### Cognitive load

To measure cognitive load, on each stage we used the scales developed by Krieglstein et al. (2023). Each scale (ICL, ECL, and GCL) consisted of 5 straightforwardly worded items (on a scale of 0 to 9). These items were selected based on theoretical definitions and subjected to factor analysis in Krieglstein et al.’s original validation study. Supplementary materials shows which items contribute to each load type. For the purposes of our study, we adjusted the questions for ECL and GCL load to ensure they could be administered after the pre-training phase and after the problem-solving phase (see Supplementary materials). Then, for each scale, the mean was calculated in line with previous studies (Krieglstein et al., 2023). The Cronbach’s alpha for the scales ranged from 0.62 to 0.92. In order to measure the overall cognitive load across stages for each type of cognitive load, scores for all questions at the pre-training stage and at the problem solving stage were summed and then means were computed (0–9). While the measurement instrument is relatively new, it addresses common psychometric limitations in cognitive load research and offers an operationalization of GCL as effort related to schema construction and elaboration.

#### Problem-solving score

The problem-solving phase consisted of six short open-ended tasks (see Supplementary materials). Of these, the first three were designed as explanation practice tasks to reinforce concept understanding and application in familiar formats, while the latter three served as problem-solving tasks, requiring learners to transfer and justify their understanding in novel contexts. This structure provides support for a scaffolded transition from guided application to independent reasoning (Margulieux and Catrambone, 2021). Although these tasks were well-structured and domain-specific, they meet established criteria for problem-solving in instructional research. Previous research emphasizes that tasks requiring strong methods are especially effective for novices (Van Merrienboer, 2013), and classifies rule-using and interpretive problems as legitimate forms of problem-solving (Jonassen, 2000). The tasks used in this study involved applying theoretical concepts, interpreting scenarios, and justifying responses: core features of early-stage problem-solving. The first three questions were graded on a scale from 0 to 2 points, while the last three questions were graded on a scale from 0 to 3 points with the maximum possible score being 15. Each score represented a discrete ordinal category based on pre-defined qualitative criteria (e.g., no justification, partial justification, complete justification). Two authors who are experts in cognitive load and instructors of the elective course conducted the assessment and marked the answers, with interrater reliability measured using Cohen’s Kappa (*κ* = 0.63–0.82), indicating moderate to substantial agreement (Conger, 1980). The final score was calculated as follows: for each student, the average score from the two raters was calculated for each question, and these averages were then summed as total score for this student.

Table 1 provides descriptive statistics for variables that were used in the analyses.

**Table 1:** Descriptive statistics for variables.

Variable	Mean	SD	Min	Max	Lower prior knowledge group		Higher prior knowledge group	
					Mean	SD	Mean	SD
Prior knowledge	6.31	1.40	2	8				
Across both stages: ICL	4.51	1.59	1	9	5.03	1.85	4.32	1.45
Across both stages: ECL	4.21	1.68	1	9	4.76	1.95	4.02	1.53
Across both stages: GCL	6.19	1.1	2.2	8.1	5.76	1.28	6.35	0.99
Pre-training: ICL	4.39	1.61	1	9	4.89	1.93	4.21	1.44
Pre-training: ECL	4.79	1.97	1	9	5.37	2.08	4.58	1.89
Pre-training: GCL	5.92	1.17	3.2	8.4	5.59	1.34	6.03	1.08
Problem solving: ICL	4.63	1.77	1	9	5.17	2.04	4.43	1.63
Problem solving: ECL	3.64	1.91	1	9	4.16	2.35	3.45	1.69
Problem solving: GCL	6.47	1.32	1.2	9	5.92	1.66	6.67	1.13
Problem-solving score	7.51	2.43	0	13.5	5.97	2.79	8.03	2.08

### Analysis strategy

To test the hypotheses, we conducted a series of repeated-measures ANOVAs separately for each type of cognitive load: ICL, ECL, and GCL. Each model included phase (pre-training vs. problem-solving) as a within-subjects factor and prior knowledge (lower vs. higher, based on a median split of prior knowledge scores) as a between-subjects factor.

Analyses were performed using the *afex* package in R (Singmann et al., 2021), which implements Type III sums of squares and is suitable for mixed designs. *Post hoc* comparisons were conducted using the *emmeans* package (Lenth, 2025), with Bonferroni adjustment for multiple comparisons.

Effect sizes for ANOVA models are reported using generalized eta squared (η^2^_G_), calculated using the *effectsize* package (Ben-Shachar et al., 2020). This allowed us to estimate the proportion of variance explained by each effect.

This analytical approach enabled us to test, first, the main effect of prior knowledge (H1); second, the main effect of phase, and finally the interaction between prior knowledge and phase (H2a, H2b), which reflects how cognitive load changes across phases depending on learners’ knowledge level.

## Results

### RQ1: what differences exist in the cognitive load experienced by learners with varying levels of prior knowledge during problem-solving after pre-training?

To test H1a–c, a repeated-measures ANOVA was conducted with phase (pre-training vs. problem-solving) as a within-subject factor and prior knowledge group (lower vs. higher) as a between-subject factor (Table 2). ICL was significantly lower for learners with higher prior knowledge than those with lower prior knowledge, *F*(1, 134) = 5.51, *p* = 0.02, *η^2^* = 0.03, supporting H1a. ECL also differed significantly between groups, *F*(1, 134) = 5.41, *p* = 0.02, *η^2^* = 0.03, consistent with H1b. GCL was significantly higher in the higher prior knowledge group, *F*(1, 134) = 8.07, *p* < 0.01, *η^2^* = 0.04, confirming H1c. These results suggest that learners with more prior knowledge experienced lower cognitive load and were able to allocate more resources to germane processes during learning (see Table 2).

**Table 2:** Main effects and interaction effects from the 2 × 2 repeated measures ANOVA on cognitive load by prior knowledge and phase.

Cognitive load type	Effect	*F*	*p*-value	*η^2^_G_*
ICL	Prior knowledge	5.51	0.02	0.03
	Phase	2.09	0.15	<0.01
	PK × phase	0.05	0.82	<0.01
ECL	Prior knowledge	5.41	0.02	0.03
	Phase	13.97	<0.01	0.03
	PK × phase	0.04	0.84	<0.01
GCL	Prior knowledge	8.07	<0.01	0.04
	Phase	2.8	0.10	<0.01
	PK × phase	1.78	0.19	<0.01

### RQ2: how does pre-training contribute to the redundancy effect for learners with different levels of prior knowledge during problem-solving?

No significant interaction effects between prior knowledge and phase were found for any type of cognitive load (all *p* > 0.19), suggesting that the patterns of change from pre-training to problem-solving did not significantly differ across groups. However, planned pairwise comparisons within each group provided more nuanced insights (Table 3). Among learners with lower prior knowledge, ECL significantly differs from pre-training to problem-solving (*p* = < 0.01), supporting H2a. GCL was higher during problem solving, but this difference did not reach a desirable level of statistical significance (*p* = 0.10), while ICL also showed a non-significant trend (*p* = 0.15). These results partially support H2a, indicating that pre-training may have facilitated learning by reducing unnecessary load.

**Table 3:** Post-hoc comparisons of cognitive load between phases (pre-training vs. problem-solving) within each prior knowledge group.

Cognitive load type	Prior knowledge group	Contrast (problem solving – pre-training)	SE	t-ratio	*p*-value
ICL	Lower	0.28	0.19	1.45	0.15
	Higher	0.23	0.12	1.96	0.05
ECL	Lower	−1.21	0.32	−3.74	<0.01
	Higher	−1.13	0.19	−5.83	<0.01
GCL	Lower	0.33	0.19	1.68	0.10
	Higher	0.63	0.12	5.38	<0.01

For learners with higher prior knowledge, ECL was also significantly lower during problem solving (*p* < 0.01), which contradicts H2b, as pre-training was expected to be redundant and lead to increased extraneous processing. Moreover, GCL was significantly higher during problem-solving (*p* < 0.01), also contrary to expectations. ICL was lower during pre-training (*p* = 0.05), partially failing to support the predicted direction of change. Overall, H2b was not supported, as higher prior knowledge learners also appeared to benefit from pre-training.

## Discussion

The present study examined the relationship between prior knowledge and cognitive load in the context of pre-training and problem-solving. The findings contribute to the discussion regarding the role of pre-training in supporting learning and cognitive load management, particularly in cases where learners may have varying levels of domain-specific knowledge (Kalyuga, 2007a, 2007b; Moreno and Mayer, 2007). While the significance of prior knowledge is well-established (Kalyuga, 2007a, 2007b; Sweller, 2024), it is underexplored how different levels of prior knowledge influence the effectiveness of pre-training interventions, which aim to introduce foundational information before complex tasks.

The first research question investigated the differences in the cognitive load of learners with lower and higher prior knowledge when they engage in problem-solving after pre-training. The results confirmed all three hypotheses (H1a, H1b, and H1c), suggesting that prior knowledge plays a crucial role in cognitive processing. As expected, learners with high prior knowledge demonstrated lower ICL which aligns with prior research stating that when learners have more domain prior knowledge and established schemas in their long-term memory, the number of interacting elements decreases (Kalyuga, 2007a, 2007b) and so does ICL.

Similarly, learners with higher prior knowledge reported lower ECL. This finding is consistent with the idea that prior knowledge has an indirect effect on ECL by allowing learners with greater domain-specific knowledge to allocate more working memory resources to overcoming the challenges posed by suboptimal instructional materials (Sweller et al., 2011). Researchers suggest that having more prior knowledge allows learners to self-manage their learning process (Castro-Alonso et al., 2021). Previous research also shows that learners with higher prior knowledge employ mental strategies to overcome some of the effects (i.e., split-attention effect) of instructional design (de Koning et al., 2020).

Finally, higher prior knowledge learners exhibited higher GCL. These findings support the assumption that learners with higher prior knowledge were able to allocate more cognitive resources to constructing and refining their schemas, a key factor in deep learning (Paas et al., 2003). The positive relationship between prior knowledge and GCL supports the argument that learners with higher prior knowledge engage in more meaningful processing strategies, allowing them to integrate new information more effectively (Leppink et al., 2014).

The second research question examined how pre-training contributes to the content redundancy effect for learners with varying levels of prior knowledge. The results provided mixed support for H2a and H2b, revealing a more nuanced relationship than initially anticipated. The retrieval of long-term memory schemas to working memory can significantly enhance effective working memory capacity by facilitating the organization of multiple information elements into a smaller number of schema-based chunks (Chen et al., 2017).

For learners with lower prior knowledge, ECL significantly decreases during the problem-solving phase compared to pre-training. However, ICL does not significantly differ between the two phases, and the increase in GCL is not statistically significant. The significant reduction of ECL during problem-solving suggests that pre-training supported learners in constructing initial schemas, which they could then draw on during problem-solving. This likely allowed them to process information more efficiently, organize and integrate new information and connect it with the content introduced during pre-training (Kalyuga, 2009), thereby alleviating the burden of poorly organized information (Retnowati et al., 2018). Rather than activating existing knowledge, pre-training may have provided the foundational cognitive structure needed for managing problem complexity.

Although a reduction in ICL and GCL was expected for learners with lower prior knowledge (Sweller et al., 2019), no significant differences were observed across phases. It can be explained by the nature of these cognitive load types. ICL arises from the inherent complexity of material defined by both element interactivity and prior domain-specific knowledge. While pre-training aims to reduce ICL by increasing relevant knowledge in long-term memory (Sweller et al., 2011), thus reducing element interactivity, it cannot eliminate completely the inherent complexity of the subsequent task. Thus, pre-training may have introduced key concepts but was insufficient to offset the full cognitive demands of the problem-solving phase. It is also important to mention that pre-training in this study consisted of two parts (concept maps and glossary) and their complexity and limited study time may have constrained its effectiveness.

Another possible explanation is that the pre-training effect tends to be most effective for learners with minimal prior knowledge (Sweller et al., 2019), which may not have applied to participants of this study. Alternatively, it is possible that learners did not fully comprehend the pre-trained material. Regarding GCL, its strong interdependence with ICL suggests that overall cognitive demands may have exceeded working memory capacity. As a result, insufficient resources may have been available for germane processing, which might have hindered effective schema construction (Ayres, 2006).

Interestingly, the redundancy effect was not strongly supported for learners with higher prior knowledge. Instead of increasing ECL, pre-training appeared to contribute to deeper learning, as evidenced by increased GCL during problem-solving. ICL remains unchanged. This suggests that pre-training may not always be redundant for these learners, which contradicts some of the previous assumptions (Akbulut et al., 2024; Kalyuga, 2007a, 2007b; Mayer and Pilegard, 2014; Trypke et al., 2023). A possible explanation is that pre-training provided an opportunity for learners with higher prior knowledge to reinforce existing schemas rather than simply reprocessing known information. These findings support previous results that show that pre-training method not only mitigates ECL but also positively impacts GCL, ultimately contributing to enhanced learning outcomes (Jung et al., 2024). While excessive instructional guidance can be detrimental, pre-training may still play a beneficial role for more experienced learners under certain conditions.

## Conclusion

This study examined the relationship between prior knowledge, pre-training, and cognitive load in the context of problem-solving, with a particular focus on how pre-training affects intrinsic, extraneous, and GCL for learners with different levels of prior knowledge. Our results indicate that pre-training can be an effective tool for reducing ECL for learners with different levels of prior knowledge, contradicting the assumption that learners with higher prior knowledge would experience redundancy due to the expertise reversal effect. However, its effect on ICL and GCL was more nuanced, suggesting that while pre-training provides foundational cognitive support, it does not fully eliminate the demands of complex problem-solving. Notably, for learners with higher prior knowledge, pre-training was not entirely redundant, as it contributed to increased GCL, implying that even more experienced learners may benefit from the opportunity to refine existing schemas under certain conditions. These findings reinforce the view that pre-training and prior knowledge activation are distinct but may interact, particularly when pre-training content overlaps with learners’ existing knowledge.

Despite these insights, several limitations should be acknowledged. First, the prior knowledge test used in this study was relatively easy, leading to an uneven distribution of participants between lower and higher prior knowledge groups. This may have impacted the ability to detect more nuanced differences between learners and should be addressed in future research by incorporating more challenging tests and other assessment tasks. However, despite this imbalance, the groups demonstrated significant differences in ICL during both the pre-training and problem-solving stages, indicating that they represent meaningfully distinct levels of prior knowledge. Second, the study used a specific pre-training format (concept maps and glossary/definitions), which may not generalize to other instructional methods. Future studies should compare different types of pre-training interventions, such as worked examples, video-based explanations, or adaptive learning environments, to determine the most effective formats for learners with varying expertise levels. Also, while the pre-training phase involved both a glossary and a concept map, we did not aim to isolate their individual effects. Future research could explore the relative contributions of these components to learning outcomes. Moreover, while pre-training can incidentally activate prior knowledge in some learners, it should be conceptually distinguished from instructional techniques that explicitly prompt knowledge retrieval. Third, although our tasks involve structured application and interpretation, they represent well-structured, rule-using problems rather than ill-structured or creative problem solving. Future studies could explore a broader range of task types to examine whether effects differ across problem types. Fourth, while the cognitive load scales used were validated and aligned with current theory (Krieglstein et al., 2023), ongoing debate regarding GCL suggests the need for continued refinement of measurement tools. Finally, the study focused on immediate learning outcomes, and long-term retention and transfer of knowledge were not assessed. Future research should include delayed post-tests to examine whether pre-training leads to sustained learning benefits over time. Future studies could also disentangle the effects of different pre-training formats (e.g., glossary vs. concept map) and compare pre-training against a control condition to better evaluate potential redundancy or benefits depending on learners’ prior knowledge levels.

In conclusion, while pre-training is generally beneficial for novice learners, its effects on experienced learners appear more variable. Moreover, the lack of reduced ICL on learners with lower prior knowledge across phases contradicts earlier findings and highlights the need for further research into the conditions under which pre-training effectively supports the management of element interactivity.

## Data Availability

The raw data supporting the conclusions of this article will be made available by the authors, without undue reservation.

## Footnotes

^1^Due to the relative ease of the prior knowledge test (mean = 6.3 out of 8), the distribution was skewed and showed a ceiling effect. As such, using prior knowledge as a continuous variable would violate assumptions of normality and lead to misinterpretation. In this context, the median was a more appropriate threshold for group assignment.
