# Supplementary figures and images for: Rethinking pre-training: cognitive load implications for learners with varying prior knowledge
Source: Front Psychol. 2025 Aug 7;16:1628047. doi: 10.3389/fpsyg.2025.1628047 (PMC12367772; doi:10.3389/fpsyg.2025.1628047)

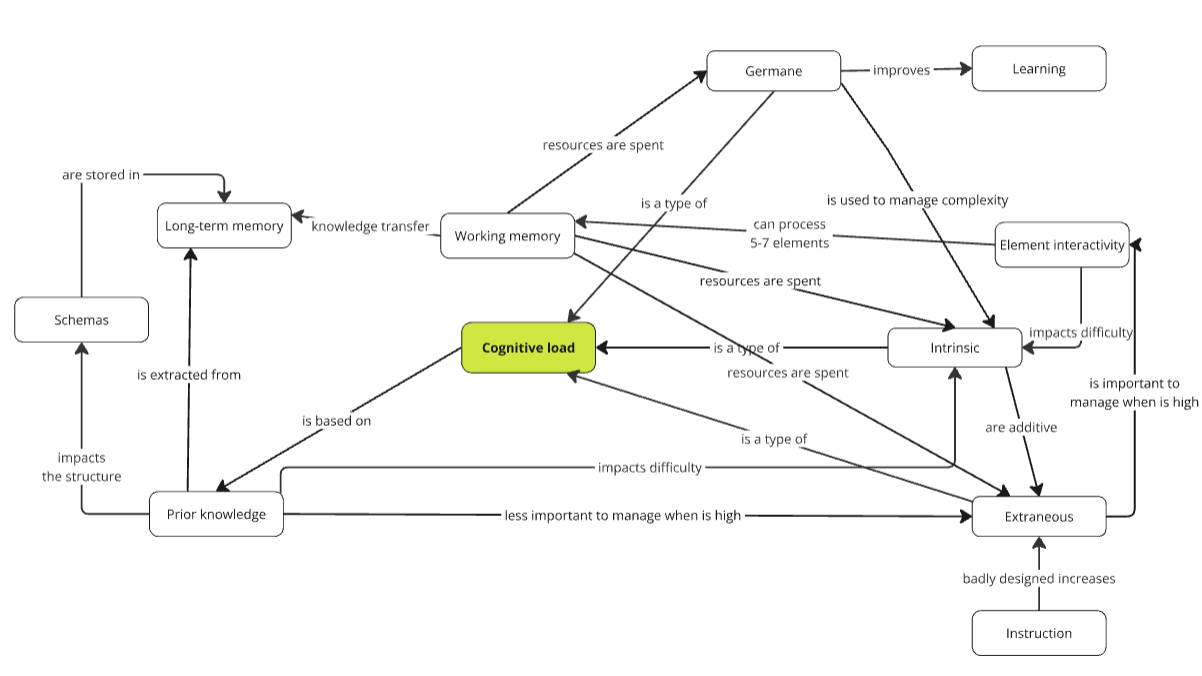

Supplement: Supplementary file 1 [file Image_1.png]
