# Supplementary material for: Rethinking pre-training: cognitive load implications for learners with varying prior knowledge
Source: Front Psychol. 2025 Aug 7;16:1628047. doi: 10.3389/fpsyg.2025.1628047 (PMC12367772; doi:10.3389/fpsyg.2025.1628047)
Supplement: Supplementary file 2 [file Presentation_1.pdf]

Supplementary Material

Glossary

Cognitive load - the amount of mental effort required to perform a task or process information
Intrinsic load - the inherent difficulty of the subject matter being learned
Extraneous load - the external factors that contribute to the complexity of the subject matter being learned
Germane load - the cognitive resources invested in the constructive and generative processing of information necessary for deep learning and understanding
Working memory - a temporary storage system for information that is currently being processed by the brain. It is responsible for holding information in the mind for a short period of time, while it is being actively processed or manipulated.
Long-term memory stores information over a longer period of time, from days to years, has no limits
Schema is a mental structure or framework that helps us organize and make sense of information. It consists of knowledge and memories about a particular subject or concept, and includes information about what the subject is like, how it behaves, and how it relates to other concepts.
Prior knowledge - information and experiences that learners have acquired before being exposed to a new concept or idea.
Learning is the process of acquiring knowledge, skills, or understanding through experience or study
Instruction is the process of teaching or providing information to a learner
Theory of learning a set of principles or ideas that attempt to explain how learning occurs.
Element interactivity: the relationships and connections between different elements within a learning task or environment.
Instructional design is the process of planning, creating, and delivering instructional materials and experiences that are designed to facilitate learning and promote understanding.
Information - raw data or stimuli that are received by the senses, such as visual, auditory, or tactile information.
Sensory memory is a type of memory that temporarily stores sensory information such as sights, sounds, and smells.
