# Supplementary material for: Rethinking pre-training: cognitive load implications for learners with varying prior knowledge
Source: Front Psychol. 2025 Aug 7;16:1628047. doi: 10.3389/fpsyg.2025.1628047 (PMC12367772; doi:10.3389/fpsyg.2025.1628047)
Supplement: Supplementary file 3 [file Presentation_2.pdf]

Supplementary Material
Microsoft form blocks

Prior knowledge test

1. What is working memory primarily responsible for?
A) Storing information permanently
B) Holding and manipulating information temporarily
C) Organizing information into long-term storage
D) Retrieving information from past experiences
E) I don't know

2. Long-term memory is characterized by:
A) Limited capacity and duration
B) Unlimited capacity and potentially unlimited duration
C) Immediate recall of information
D) The ability to learn through observation
E) I don't know

3. A schema helps a learner to:
A) Memorize facts without understanding
B) Make connections between new information and existing knowledge
C) Ignore irrelevant information entirely
D) Techniques for memorizing information
E) I don't know

4. Cognitive load theory suggests that:
A) Learning is best achieved through passive observation.
B) Too much information can overwhelm working memory and hinder learning.
C) All types of cognitive load are beneficial for learning.
D) Long-term memory has no impact on cognitive load.
E) I don't know

5. What is the primary purpose of memory in the learning process?
A) To store information indefinitely
B) To retrieve information when needed
C) To manipulate information in real-time
D) Both B and C
E) I don't know

6. Prior knowledge plays a crucial role in learning because it:
A) Has no impact on how new information is understood
B) Can create misconceptions that hinder learning
C) Helps learners connect new information to existing frameworks

D) Only benefits advanced learners
E) I don’t know

7. Which of the following best describes extraneous cognitive load?
A) The mental effort required to learn new information that is essential for understanding the material.
B) *The mental effort that is unnecessary and arises from poorly designed instructional materials or irrelevant information.*
C) The mental effort involved in recalling previously learned information during a test.
D) The mental effort required to manage emotions and motivation while learning.
E) I don’t know

8. Which of the following principles is most important when designing effective instructional materials?
A) Using as much text as possible to ensure thorough explanations.
B) *Incorporating multimedia elements that align with the learning objectives and enhance understanding.*
C) Focusing solely on the aesthetics of the materials without considering content.
D) Providing information in a single format to avoid overwhelming learners.
E) I don’t know

Cognitive load questions after pre-training phase

Item	Cognitive Load Type
I found it difficult to understand Cognitive Load Theory.	Intrinsic Load
It was difficult to understand the structure of the concept map.	Intrinsic Load
I thoroughly engaged with Cognitive Load Theory.	Germane Load
The explanation of Cognitive Load Theory was difficult to understand.	Intrinsic Load
The design of the concept map made it difficult to understand the relationships between different pieces of information.	Extraneous Load
I made an effort to understand Cognitive Load Theory.	Germane Load
Cognitive Load Theory is complex.	Intrinsic Load
The design of the concept map was inconvenient.	Extraneous Load
I fully mastered the information about Cognitive Load Theory.	Germane Load
Cognitive Load Theory contains a lot of complex information.	Intrinsic Load
The design of the concept map made it difficult to quickly find the necessary information.	Extraneous Load
I was able to expand my knowledge through the information about Cognitive Load Theory.	Germane Load
Without prior knowledge, it was difficult to understand Cognitive Load Theory.	Intrinsic Load

The design of the concept map made me feel like I couldn't focus on Cognitive Load Theory.	Extraneous Load
I can quickly and accurately apply the knowledge gained from studying concept maps.	Germane Load

Problem solving

1. Explain in your own words what you understand by cognitive load.
2. What, in your understanding, is the main difference between intrinsic and extraneous cognitive load?
3. Which type of cognitive load do you think is affected by the organization of instructional materials? Justify your answer.
4. Imagine you are navigating to an unfamiliar café from the metro using your phone’s map app. You have 15 minutes left to walk, but your phone’s battery is critically low and will shut off in 3 minutes. You need to memorize the route to avoid getting lost. What type of memory will be engaged? List the main characteristics of this type of memory using your own words.
5. Imagine you are studying a chemical reaction between two substances – Substance A and Substance B – resulting in the formation of a new substance, C. The reaction requires a specific set of conditions, including a particular temperature and the presence of a catalyst. During a laboratory session, you need to simultaneously monitor all these conditions.
   - Which two concepts from cognitive load theory can explain the processing of this information?
   - What type of cognitive load is involved in this case?

6. Consider two types of instructional materials for learning about the human respiratory system:

a) The respiratory system consists of the lungs, trachea, bronchi, and alveoli. The lungs are the primary respiratory organs located in the chest cavity. The trachea, or windpipe, is a tube that carries air from the mouth and nose to the lungs. The trachea branches into the bronchi, which further divide into smaller bronchioles leading to the alveoli—tiny air sacs where gas exchange between air and blood occurs.

b)

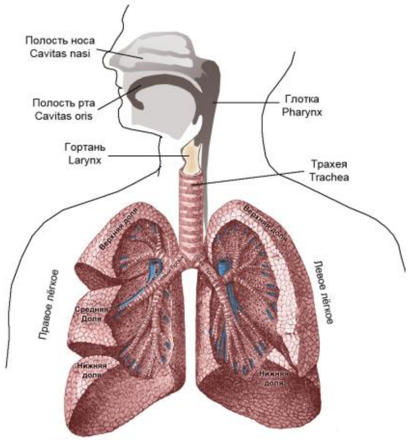

- Which material do you think increases cognitive load?
- What type of load does it increase?
- Why?

Cognitive load questions after problem solving phase

Item	Cognitive Load Type
I found it difficult to understand Cognitive Load Theory.	Intrinsic Load
It was difficult to understand the structure of the tasks.	Intrinsic Load
I thoroughly engaged with the tasks.	Germane Load
The explanation of Cognitive Load Theory was difficult to understand.	Intrinsic Load
The design of the tasks made it difficult to understand the relationships between different pieces of information.	Extraneous Load
I made an effort to complete the tasks.	Germane Load
Cognitive Load Theory is complex.	Intrinsic Load
The design of the tasks was inconvenient.	Extraneous Load
I fully mastered the information about Cognitive Load Theory.	Germane Load
Cognitive Load Theory contains a lot of complex information.	Intrinsic Load
The design of the tasks made it difficult to quickly find the necessary information.	Extraneous Load
I was able to expand my knowledge through the information about Cognitive Load Theory.	Germane Load
Without prior knowledge, it was difficult to understand Cognitive Load Theory.	Intrinsic Load
The design of the tasks made me feel like I couldn't focus on Cognitive Load Theory.	Extraneous Load
I can quickly and accurately apply the knowledge gained from completing the tasks.	Germane Load
